# Suppression of Gene Juvenile Hormone Diol Kinase Delays Pupation in *Heortia vitessoides* Moore

**DOI:** 10.3390/insects10090278

**Published:** 2019-09-02

**Authors:** Zihao Lyu, Zhixing Li, Jie Cheng, Chunyan Wang, Jingxiang Chen, Tong Lin

**Affiliations:** College of Forestry and Landscape Architecture, South China Agricultural University, Guangzhou 510642, China

**Keywords:** juvenile hormone diol kinase, juvenile hormone, *Heortia vitessoides* Moore, RNA interference, triglyceride

## Abstract

Juvenile hormone diol kinase (JHDK) is a critical enzyme involved in juvenile hormone degradation in insects. In this study, *HvJHDK* in the *Heortia vitessoides* Moore (Lepidoptera: Crambidae) transcriptional library was cloned. Stage-specific expression patterns of *HvJHDK*, *HvJHEH*, and *HvJHE* as well as juvenile hormone titers were determined. The three tested enzymes participated in juvenile hormone degradation. Moreover, juvenile hormone titers peaked after larval–larval molts, consistent with a role for juvenile hormone in inhibition of metamorphosis. *HvJHDK* was subsequently suppressed using RNA interference (RNAi) to reveal its functions. Different concentrations of ds*JHDK* elicited the optimal interference efficiency at different life stages of *H. vitessoides*. Suppression of *HvJHDK* decreased HvJHDK content and increased the juvenile hormone titer, thereby resulting in reduced triglyceride content, sharply declined survival rate, clearly lethal phenotypes, and extended larval growth. Moreover, suppression of *HvJHDK* upregulated *HvJHEH* and *HvJHE* expression levels, suggesting that there is feedback regulation in the juvenile hormone metabolic pathway. Taken together, our findings provide molecular references for the selection of novel insecticidal targets.

## 1. Introduction

Juvenile hormone (JH) is one of the most important insect hormones. This unique sesquiterpenoid hormone is crucial for various physiological processes in insects, including embryonic development, metamorphosis regulation, imaginal disc formation, sexual maturation, reproduction, pheromone production, and nutrition [1,2,3,4,5]. JH is mainly produced in the corpus allatum and released into the hemolymph, and through the hemolymph, JH is then transferred to target tissues via the carrier juvenile hormone-binding protein [6]. To date, seven natural JHs have been identified, and of those, JH III plays a primary physiological role [7,8]. In insects, JH synthesis and metabolism rates are tightly coupled with the JH titer balance [9,10]. Biosynthesis of JH involves the isoprenoid branch of the mevalonate (MVA) pathway. Biological functions of JH are realized when it binds to the heterodimeric receptor methoprene-tolerant (MET) and the nuclear receptor USP to induce protein kinase C signaling and calcium signal transduction [11]. At least three enzymes, namely, juvenile hormone esterase (JHE; EC 3.1.1.1) [12], juvenile hormone epoxide hydrolase (JHEH; EC 3.3.2.3) [13], and juvenile hormone diol kinase (JHDK; EC 2.1.7.3) [14], catalyze JH metabolism [15]. JHE converts JH to JH acid (JHa) via methyl ester hydrolysis, and JHEH hydrolyzes the JH epoxide moiety to produce JH diol (JHd). Alternatively, JHE catalyzes JHd, and JHEH degrades JHa to produce JH acid diol (JHad) [13]. In insects, cytoplasmic JHDK catalyzes JHd to JH diol phosphate (JHdp). Currently, JHad and JHdp are considered the principal final metabolites of JH degradation [14]. JHDK was first identified by Maxwell and colleagues, who isolated and purified *Manse*-JHDK from the Malpighian tubules of larval *Manduca sexta*. *Manse*-JHDK is active as a homodimer, is specific to JHd, prefers ATP over GTP, and requires Mg^2+^ for its activity; however, low concentrations of calcium ions inhibit *Manse*-JHDK activity [14]. Maxwell et al. performed cDNA cloning and confirmed that *Manse*-JHDK possessed elongation factor (EF)-hand motifs, as revealed by the amino acid sequence; moreover, they reported that three conserved sequence elements were involved in purine nucleotide binding, which were similar to GTP-binding proteins. To date, the majority of the studies on JH-metabolizing enzymes have focused on JHE and JHEH [16,17,18,19,20,21]. However, information regarding the roles of JHDK in the regulation of JH titers remains limited. In the present study, RNA interference (RNAi) of *JHDK* is reported for the first time in *Heortia vitessoides* Moore (Lepidoptera: Crambidae) to reveal the role of *JHDK* in JH degradation in insects. The findings of this study can be useful in the selection of novel insecticidal targets.

*H. vitessoides* is a serious defoliator of the evergreen tree *Aquilaria sinensis* in tropical and subtropical regions. This tree has medicinal properties and provides raw material for the production of incense, which has enormous economic value. When *A. sinensis* is severely infested, *H. vitessoides* completely denudes the leaves [22], which leads to substantial financial loss. A comprehensive molecular understanding of *H. vitessoides*—a holometabolous insect—is thus warranted to help control this pest.

## 2. Materials and Methods

### 2.1. Insects

*H. vitessoides* was collected from Tianlu Lake Forest Park, Guangzhou and reared in a climatic cabinet at 26 °C with 70–75% relative humidity and a 14 L:10 D photoperiod. *H. vitessoides* larvae were fed with *A. sinensis* leaves.

Mature larvae were transferred to a silt basin for harvesting pupae and adults. Newly emerged adults were fed with 7% honey solution. *H. vitessoides* eggs, 1-day-old first to 1-day-old third molting instars, 12-h-old fourth to 168-h-old fifth instars, 1- to 9-day-old pupae, and adults were collected to examine the stage-specific expression profiles of *HvJHDK*, *HvJHEH*, and *HvJHE*. The collected insects were immediately snap-frozen in liquid nitrogen and stored at −80 °C until further use.

### 2.2. RNA Extraction and cDNA Synthesis

Total RNA was extracted from each sample using the E.Z.N.A.^TM^ Total RNA Kit II (Omega Bio-Tek, Norcross, GA, USA), following the manufacturer’s instructions. First-strand cDNA was synthesized from 2 μg of total RNA from each sample using the PrimeScript^®^ RT Reagent Kit with gDNA Eraser (Takara Bio, Otsu, Japan) and immediately stored at −20 °C until further use.

### 2.3. Sequence Characterization and Phylogenetic Tree Analysis

Based on the *H. vitessoides* transcriptome (SRX4045498), a putative unigene cDNA encoding JHDK was obtained. Open Reading Frame (ORF) Finder was used to acquire the cDNA sequence of *HvJHDK* ORF. Then, the corresponding pair of gene-specific primers (Table S1) was designed to amplify the *HvJHDK* ORF to verify the sequence. The GenBank accession numbers are listed in Table S2. PCR was conducted as follows: 5 min at 95 °C; 34 cycles of 30 s at 95 °C, 30 s at 58 °C, and 2 min at 72 °C; and 10 min at 72 °C. Next, the PCR product was gel-purified, ligated into the pClone007 simple vector (TSINGKE Bio, Guangzhou, China), transformed into *Escherichia coli* DH5α competent cells (Takara Bio, Otsu, Japan), and sequenced (TSINGKE Bio, Guangzhou, China). The identities of the recovered cDNAs of *H. vitessoides* were confirmed using BLASTx. JHDK amino acid sequences of other insect species were retrieved from the National Center for Biotechnology database. DNAMAN 6.0 (LynnonBiosoft, Quebec, QC, Canada) was used to edit sequences and perform multiple sequence alignment. Physicochemical properties of HvJHDK were predicted using ProtParam, and organization of domains in the sequences was predicted using SMART. A phylogenetic tree was constructed with MEGA-X and ClustalX using the neighbor-joining method.

### 2.4. Synthesis of Double-Stranded (ds) RNA Targeting HvJHDK and Injection of dsRNA

All reagents for dsRNA synthesis in the RNAi experiments were obtained from the T7 RiboMAX^TM^ Express RNAi System Kit (Promega, Madison, WI, USA). Primers harboring T7 RNA polymerase promoter sequences were designed for PCR to obtain DNA templates. Primers used to synthetize dsRNA are listed in Table S1. DNA templates for *HvJHDK* and green fluorescent protein were used for a transcription reaction with T7 RNA polymerase to generate ds*JHDK* (258 bp) and ds*GFP* (400 bp) fragments. ds*GFP* was used as a negative control for nonspecific effects of dsRNA. The DNA template was removed, followed by dsRNA annealing and single-stranded RNA (ssRNA) removal through nuclease digestion. Next, the dsRNA was purified according to the manufacturer’s protocol (Promega). After purification, the dsRNA was dissolved in nuclease-free water, quantified using a NanoDrop 2000 spectrophotometer (Thermo Fisher Scientific, Waltham, MA, USA), and confirmed using 1.5% agarose gel electrophoresis to ensure purity and integrity. All samples were stored at −80 °C until further use.

To determine the optimal efficiency of ds*JHDK* RNAi, different concentrations of ds*JHDK* were injected into 12-h-old fourth (L4-injected group) and fifth instar (L5-injected group) larvae. The ds*JHDK* solution was diluted to 1, 2, 3, 4, and 5 μg/μL for injection. The volume injected per larva was 1 μL. Test insects were injected at the lateral internode membrane of the seventh and the eighth segments using FemtoJet (Eppendorf, Hamburg, Germany). The same concentration and volume of ds*GFP* and blanks were used as controls. A total of 30 larvae each were included in the treatment and the control groups to determine the interference efficiency of ds*JHDK*. A total of 40 larvae each were included in the L4-injected group, the L5-injected group, the ds*GFP*-injected group, and the CK (blank control, without any treatment) group to determine L4/L5 survival rate, pupation rate, and adult emergence rates, and experiments were repeated independently three times (consisting of 40 insects).

### 2.5. Quantitative Real-Time PCR (RT-qPCR)

Total RNA was extracted from each sample. First-strand cDNA was synthesized using the PrimeScript RT Reagent Kit with gDNA Eraser (Takara Inc., Dalian, China). The synthesized cDNA was then used as a template for RT-qPCR to verify the specificity and amplification efficiency of primers. Primer 5.0 was used for primer designing. Primers used for PCR are listed in Table S1. *α-tubulin* [23] was used as an internal reference. The reaction system comprised 20 μL cDNA template, 10 μL TB Green™ Premix ExTaq™ (Takara Inc.), 0.4 μL forward primer, 0.4 μL reverse primer, and 7.2 μL ddH_2_O. Non-template reactions (replacing cDNA with diethyl pyrocarbonate-treated water) were used as negative controls. Reaction conditions included initial denaturation at 95 °C for 5 min, followed by 40 cycles at 95 °C for 10 s and 60 °C for 20 s. All the reactions were run using a LightCycler^®^ Real-Time PCR System (Roche Diagnostics, Indianapolis, IN, USA). Relative expressions for each sample were calculated using the 2^−△△Ct^ method [24]. Three biological replicates and three technical replicates were run for RT-qPCR analysis [25,26,27,28,29,30].

### 2.6. Analysis of HvJHDK Protein Concentration

After 36, 60, 84, and 108 h of dsRNA injection, the insects were prepared for protein content assays as follows. Surface dirt was removed with aseptic water, the insects were weighed, phosphate buffered saline (PBS) was added, and the insects were homogenized by hand. The samples were then centrifuged at 2000–3000 rpm for 20 min to separate the supernatant.

The Insect JHDK ELISA Kit (Shanghai Enzyme-linked Biotechnology Co., Ltd., Shanghai, China) was used to measure the JHDK protein concentrations. Purified insect JHDK antibody was used to coat microtiter plate wells and produce a solid-phase antibody. Then, samples were added to the wells, and the antibody was labeled with horseradish peroxidase (HRP) to form an antibody–antigen–enzyme–antibody complex. After thorough washing, 3,3′,5,5′-tetramethylbenzidine (TMB) substrate solution was added; TMB substrate turns blue when catalyzed by HRP. The reaction was terminated by adding sulfuric acid, and the color change was measured at 450 nm using the Varioskan LUX Multimode Microplate Reader (Thermo Fisher Scientific, Waltham, MA, USA).

The total JHDK concentration in the samples was determined by extrapolating the optimal density of the samples on the linear regression equation of the standard curve.

### 2.7. Analysis of Triglyceride (TG) Content

The lipid of the insect is mainly in the form of triglyceride (TG). To identify the TG content, living 5-day-old fifth instar larvae and 1-day-old pupae injected with ds*JHDK* were collected and homogenized. The homogenates (10% *w*/*v*) were centrifuged at 2500 rpm (4 °C) for 10 min, and the supernatant was stored at 4 °C until analysis. PBS (0.1 mol/L, pH 7.4) was used as the homogenate medium. TG content was determined using a triglyceride assay kit (Nanjing Jiancheng Bioengineering Institute, Nanjing, China) following the manufacturer’s instructions. Sample optical density (OD) values were determined by measuring absorbance at 510 nm using the Varioskan™ LUX multimode microplate reader (Thermo Fisher Scientific). The TG content was normalized with the total protein content, which was determined using the Bradford Protein Assay Kit (Nanjing Jiancheng Bioengineering Institute, Nanjing, China). Three independent replicates were used for the assays.

### 2.8. Measurement of JH Titer

The JH titer of *H. vitessoides* was determined using reversed-phase high-performance liquid chromatography (RP-HPLC) following the method reported by Zhang [20]. After accurately weighing them, the insects were homogenized in methanol:diethyl ether at an equal volume ratio, followed by ultrasonic crushing of the homogenate for 10 min to completely extract JH. Next, 2 mL n-hexane was added, followed by ultrasonic oscillation at 4 °C. The supernatant was centrifuged at 12,000 rpm for 10 min. Extraction was repeated thrice, and the supernatants were combined. The resulting supernatant was dried by blowing under high purity nitrogen. After drying, the supernatant was dissolved in 500 μL chromatography grade methanol, filtered through a 0.22 μm organic membrane, sealed in a 2 mL sampling bottle, and stored at −20 °C until use. The mobile phase used was 80% methanol (methanol:water = 80:20) at a mobile phase flow rate of 0.8 mL/min, UV detector wavelength (λ) of 218 nm, and sample volume of 20 μL. LC20AD (Shimadzu, Shanghai, China) Zorbax Extend-C18 (Agilent, Beijing, China) reversed-phase column (4.6 × 250 mm) was used. The JH III standard was purchased from Sigma. The JH titers in each sample were calculated by extrapolating the values on the linear regression equation of the standard curve. Three independent biological replicates were used for the assays.

### 2.9. Phenotype Analysis

The injected individuals were checked frequently and carefully for possible phenotypic changes. The insects that did not react to brush touching within a minute were regarded as dead. In this species, the initiation of pupation is indicated by formation of a pupal chamber. To check for pupation, a small slit was made in the pupal chamber with a blade. The living individuals were continuously kept in soil to check for adult emergence. The emergence rates were evaluated during a 3 week observation period.

### 2.10. Statistical Analysis

Gene expression data are presented as the means ± standard deviation (SD) for three independent replicates. Statistical package for the social sciences v18 (SPSS; IBM, NewYork, NY, USA) was used for one-way analysis of variance and Duncan’s multiple comparisons. A Student’s t-test was performed to compare the different expressions between the controls and the treated individuals. GraphPad Prism 5 was used to create figures.

## 3. Results

### 3.1. HvJHDK Characterization and Phylogenetic Analysis

**Figure 1:**
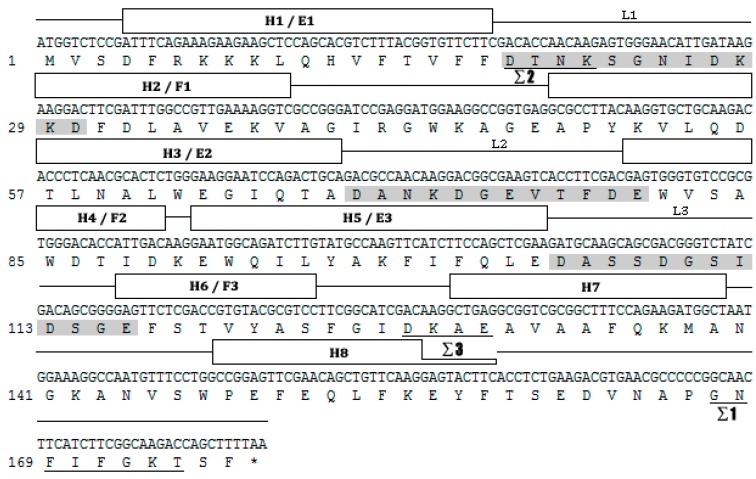
Nucleotide and deduced amino acid sequences of juvenile hormone diol kinase *Heortiavitessoides* (*HvJHDK*). The three potential fingerprint motifs (i.e., Σ1–3) of GTP-binding proteins are underlined, and the three elongation factor (EF)-hand motifs (calcium-binding motifs) are presented in shaded boxes. The α-helical sections (H1–8) are boxed and connected with lines, indicating loops or unstructured regions. E1–L1–F1, E2–L2–F2, and E3–L3–F3 form three putative EF hands, i.e., helix–loop–helix structural domains.

**Figure 2:**
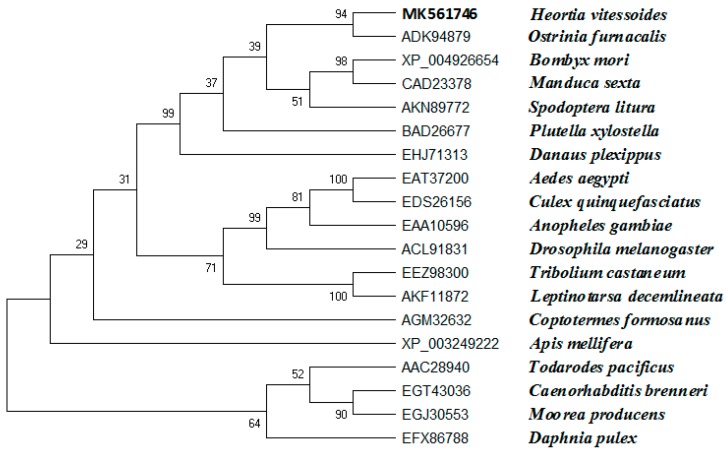
Phylogenetic analysis of juvenile hormone diol kinase homologs from 19 insect species based on amino acid sequences. A phylogenic tree was constructed using MEGA-X with sequences obtained from GenBank. Bootstrap analysis results of 1000 replicates are shown.

Based on the *H. vitessoides* transcriptome, the putative cDNA sequence of *JHDK* was obtained and designated as *HvJHDK* (GenBank: MK561746). This sequence was 531 bp long and encoded a 176 amino acid protein with a theoretical molecular weight of 19.83 kDa and an isoelectric point of 4.60. The instability index of the protein was 22.4, indicating that it was stable. Three predicted GTP-binding sites and three predicted calcium-binding motifs, which are highly conserved among JHDK proteins, were present in the deduced protein sequence (Figure 1) [8,31]. Sequence alignment using DNAMAN revealed that *HvJHDK* had a high sequence similarity with other insect *JHDKs*. A phylogenetic tree was constructed to display the relationship among insect JHDKs (Figure 2). Both *HvJHEH* and *HvJHE* were identified in the same way.

### 3.2. RNAi Efficiency for HvJHDK

**Figure 3:**
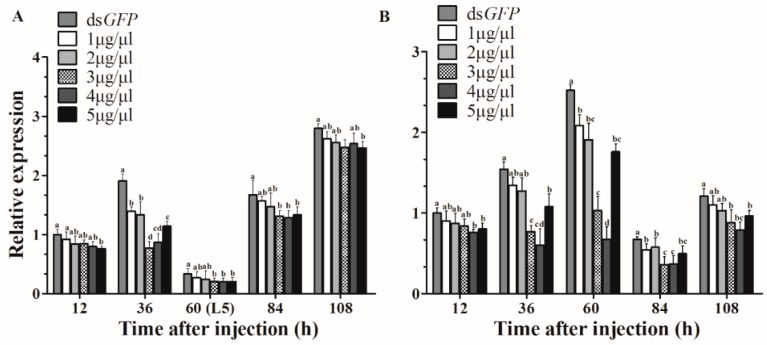
Assessment of the efficiency of RNA interference. (**A**) Relative expression levels of *HvJHDK* in fourth instar larvae after injection of doses of 1, 2, 3, 4, and 5 μg/μL. (**B**) Relative expression levels of *HvJHDK* in fifth instar larvae after injection of doses of 1, 2, 3, 4, and 5 μg/μL. Different letters indicate significant differences at *p* < 0.05 according to one-way analysis of variance (ANOVA). The data represent the mean ± SD (n = 3).

As expected, compared with control expression, *HvJHDK* expression was knocked down after injection. A concentration of 3 μg/μL ds*JHDK* showed the highest RNAi efficiency 36 h after injection in the L4-injected group, with an optimal interference of approximately 60% (Figure 3A). A concentration of 4 μg/μL ds*JHDK* showed the highest RNAi efficiency 60 h after injection in the L5-injected group, with an optimal interference of approximately 73% (Figure 3B). Based on these findings, we continued injecting concentrations of 3 and 4 μg/μL into 12-h-old L4 and L5 larvae, respectively, as follow-up experiments.

**Figure 4:**
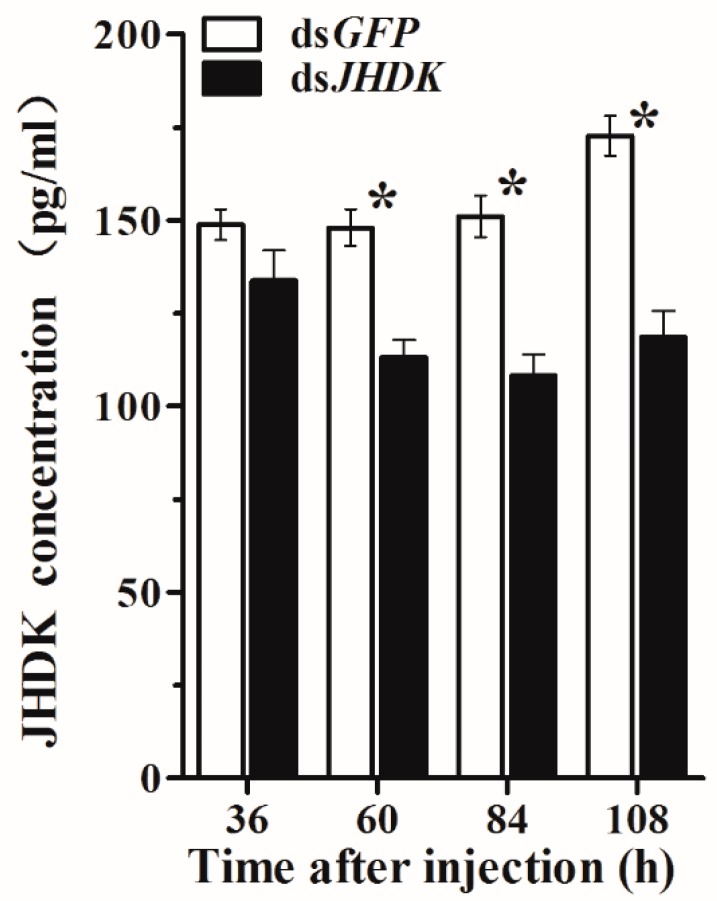
Changes in HvJHDK concentration after injection in L5-injected individuals. The data represent the mean ± SD (n = 3). The asterisk (*) indicates a significant difference according to Student’s t-test (*p* < 0.05).

After 36, 60, 84, and 108 h of injection in the L5-injected group, we assessed the HvJHDK concentration in the whole body of the insects. The HvJHDK concentration was significantly reduced in all cases except after 36 h of injection (Figure 4). These results substantiated the success of RNAi by ds*JHDK* injection in terms of protein translation.

### 3.3. JH titers and Developmental Stage-Specific Expression of HvJHDK, HvJHEH, and HvJHE

**Figure 5:**
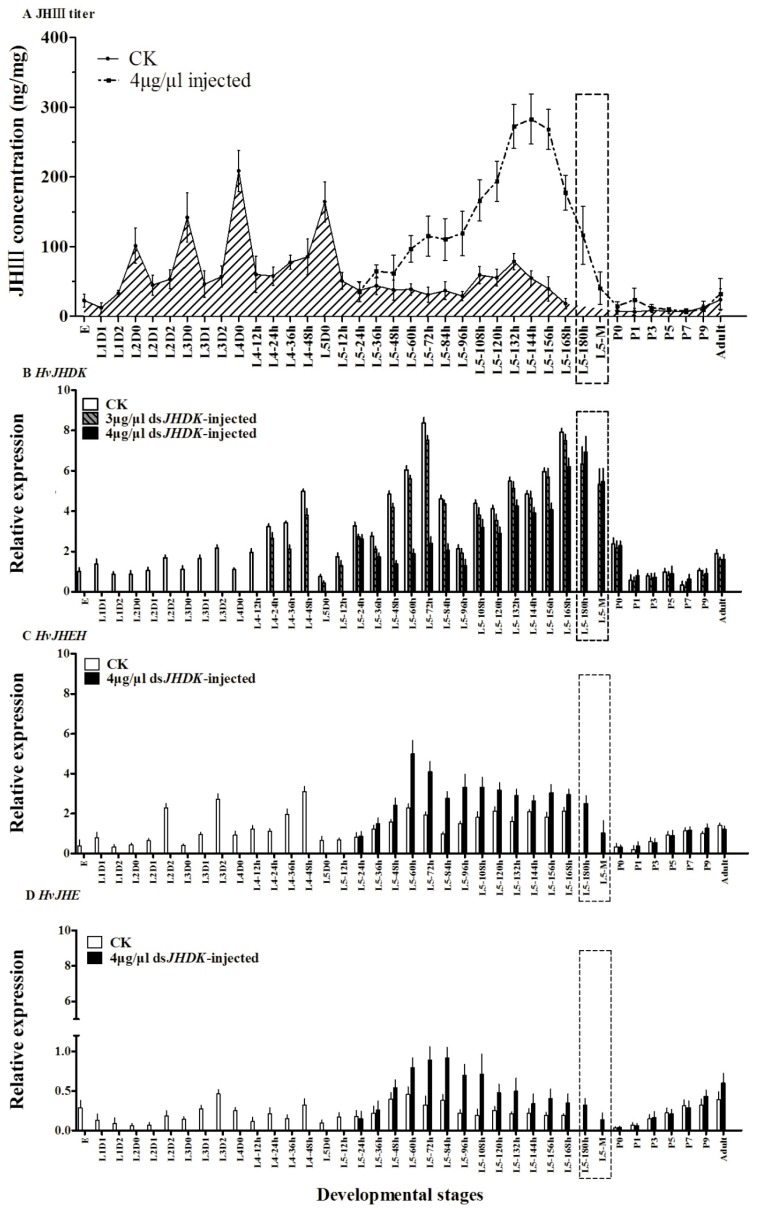
Juvenlie hormone (JH) titer (**A**) and expression profiles of *HvJHDK* (**B**), juvenile hormone epoxide hydrolase *(JHEH)* (**C**), and juvenile hormone esterase *(JHE)* (**D**) at different development stages. E, eggs; L1D1-L4D0, 1-day-old first–fourth instar larvae immediately after molting; L4-12h-L5-168h, 12-h-old fourth to 168-h-old fifth instar larvae; P0–P9, pupae immediately after molting to 9-day-old pupae; A, adults. Extended final larval stages are marked using square frames. CK, blank control. The data represent the mean ± SD (n = 3).

In this study, we determined the JH titers at different development stages of *H. vitessoides* (Figure 5). From the first to the fourth instar stages, the JH titers peaked immediately after molting. The highest peak at L4D0 reached a value of 208.5 ng/mg. In contrast, the JH titer was the lowest at the final stage of molting, which is consistent with its function of inhibiting metamorphosis. The JH titer was upregulated after the suppression of its degradation gene *HvJHDK*.

*HvJHDK*, *HvJHE*, and *HvJHEH* were consistently expressed at all tested developmental stages (Figure 5). The highest peak of *HvJHDK* expression was detected in 72-h-old fifth instar larvae. However, at all stages, the expression of *HvJHE* was relatively lower than those of *HvJHEH* and *HvJHDK*. The expression of *HvJHEH* was lower than that of *HvJHDK* at all stages. Following suppression of *HvJHDK*, both *HvJHE* and *HvJHEH* were upregulated.

### 3.4. Phenotype Analysis after RNAi

**Figure 6:**
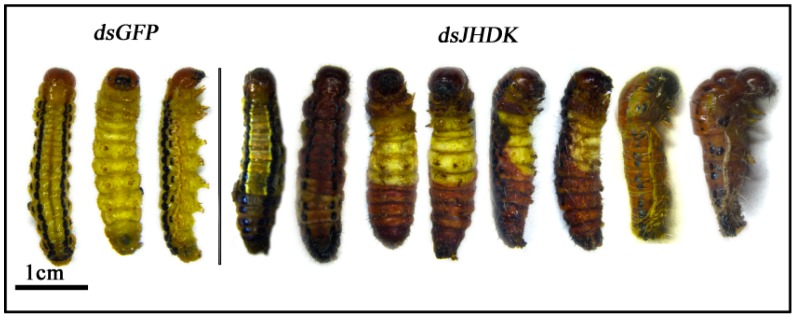
Lethal phenotypes caused by RNA interference for *HvJHDK.*

**Figure 7:**
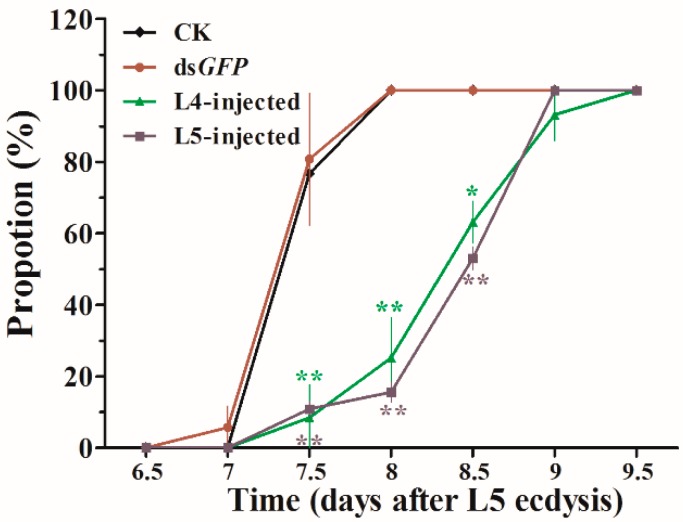
Loss of HvJHDK function resulted in extended larval periods. Proportion refers to the percentages of larvae in the different treatment groups that ecdyzed to the pupal stage. Dead larvae were not included in the statistical analyses. Data are presented as mean ± SD (n = 3). The asterisk indicates a significant difference according to Student’s t-test (* *p* < 0.05; ** *p* < 0.01).

Representative phenotypes of the larvae after ds*JHDK* and ds*GFP* injections were examined (Figure 6). Obvious abnormal/lethal phenotypes, i.e., darkened body color and insects trapped in the old cuticle during molting, were observed. The total developmental duration of L5-injected insects from the fifth instar stage to pupation increased from 7.5 to 8.5 days. The rate of growth is presented in Figure 7. Pupal weight slightly increased in the treatment group compared to the control groups (Table 1).

### 3.5. Relative Gene Expression after RNAi

**Figure 8:**
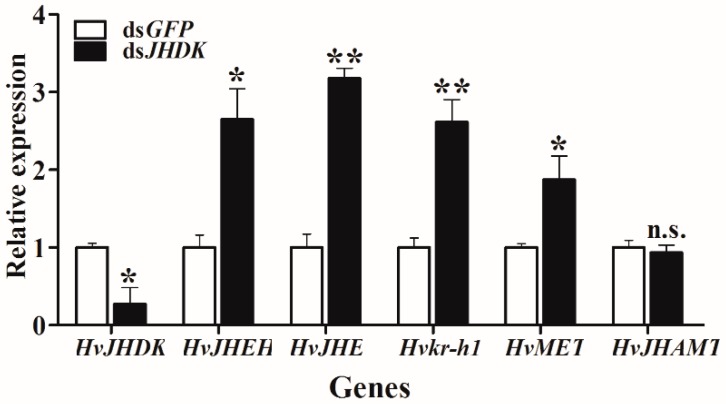
Relative gene expression in *dsJHDK*-injected larvae versus ds*GFP*-injected larvae. Expression levels of the JH metabolic and response genes *HvJHDK*, *HvJHEH*, *HvJHE*, *JH acid methyltransferase* (*HvJHAMT)*, *HvMET*, and *Krüppel homolog 1 (Hvkr-h1)* were investigated 72 h after injection of dsRNA. The data represent the mean ± SD (n = 3). The asterisk indicates a significant difference according to Student’s t-test (* *p* < 0.05; ** *p* < 0.01; n.s. not significant).

We tested the relative gene expression after dsJHDK injection at 72 h via RT-qPCR. As shown in Figure 8, the expressions of *HvJHEH*, *HvJHE*, *Krüppel homolog 1* (*Hvkr-h1*), and *HvMET* were increased, while there was no significant change in the expression level of *JH acid methyltransferase* (*HvJHAMT*).

### 3.6. Analysis of TG Content and the Expression of Lipid Accumulation and Degradation-Related Genes

**Figure 9:**
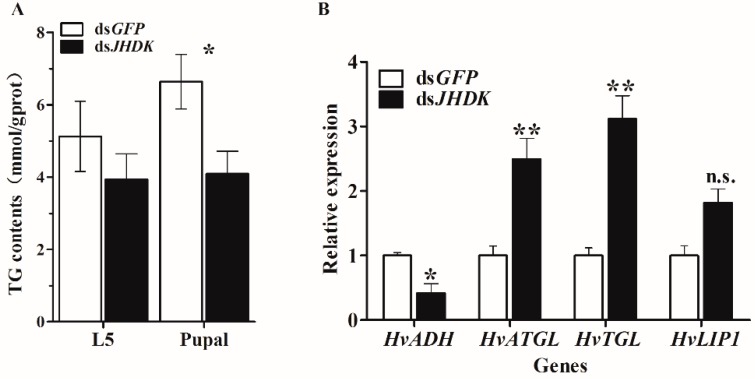
Analysis of triglyceride (TG) content. (**A**) Assessment of TG content in fifth instar larvae and pupae after injection at 108 h and 204 h, respectively. The data represent the mean ± SD (n = 3), each repetition contained 10 individuals. (**B**) Relative gene expression of the lipid accumulation and the degradation-related genes *alcohol dehydrogenase* (*HvADH*), *adipose triglyceride lipase* (*HvATGL*), *triglyceride lipase* (*HvTGL*), and *lipase 1* (*HvLIP1*) 72 h after injection of dsRNA. The data represent the mean ± SD (n = 3). The asterisk indicates a significant difference according to Student’s t-test (* *p* < 0.05; ** *p* < 0.01; n.s. not significant).

We tested the TG content after ds*JHDK* injection at 108 h and 204 h, respectively, to identify the changes in TG content induced by suppression of *HvJHDK*. The results showed that the TG content of the insect decreased at 108 h and 204 h after injection of ds*JHDK*. We also tested the expression of lipid accumulation and degradation-related genes after ds*JHDK* injection at 72 h via RT-qPCR. The results showed that the expression of *alcohol dehydrogenase (HvADH)* gene was decreased, and the expression of *adipose triglyceride lipase* (*HvATGL*), *triglyceride lipase* (*HvTGL*), and *lipase 1* (*HvLIP1*) genes were increased (Figure 9).

## 4. Discussion

*JHDK* is an important enzyme that metabolizes JH in insects. In this study, a novel cDNA sequence of *JHDK* was designated from the *H. vitessoides* transcriptional library. Homologous alignment and phylogenetic tree analyses revealed that *HvJHDK* is highly homologous with JHDKs in other insect species. The well conserved GTP-binding protein motifs and calcium-binding sites of JHDKs [6,31] were present in the predicted protein sequence of HvJHDK. These results suggest that HvJHDK is highly similar to JHDKs in other insects.

To explore the functions of JHDK using RNAi, we first determined the optimal interference concentration. It was necessary to determine the optimal time point and concentration for application for the following RNAi experiments. We found that the sensitivity of the fourth and the fifth instar larvae to ds*JHDK* differed, and that injections of 3 and 4 μg/μL of ds*JHDK* produced the optimal interference efficiency in fourth and fifth instar larvae, respectively. The interference effect was found to last for approximately 180 h (including extended final larval stage) in the L5-injected larvae (4 μg/μL). The interference efficiency of ds*JHDK* (<10%) recovered faster in the L4-injected larvae (3 μg/μL), with the effects lasting for approximately 108 h. (Figure 5B). The expression levels of the introduced corresponding genes of dsRNA are generally believed to return to the original level after being degraded. Furthermore, both L4- and L5-injected larvae showed lower adult emergence rates than pupation and L4–L5 survival rates. In *H. vitessoides*, the adult emergence process might be more dependent on HvJHDK for it to exert its effects. Abnormal elevation of the JH titer inhibiting the growth of imaginal discs may be one reason underlying the low adult emergence rates [2].

JH plays an important role in regulating growth and development in larval insects, and JH degradation is specific to developmental stages, coinciding with its functions at each stage [32]. In the present study, we systematically assessed the expression patterns of the three JH degradation genes *HvJHDK*, *HvJHEH*, and *HvJHE* and measured the JH titers at different developmental stages. These developmental profiles are invaluable because the timing of changes in the JH titers (HvJHDK induction) may correlate with changes in the status of physiological events [33]. The JH titers in *H. vitessoides*, which vary with developmental stages, peaked immediately after molting (L2D0, L3D0, L4D0, and L5D0), and JH secretion gradually decreased at the end of the fifth instar stage, consistent with its role in metamorphosis inhibition. Similar to results obtained in *Helicoverpa armigea* [20], the expression of *HvJHDK* (L3D0, L4D0, and L5D0), *HvJHEH* (L3D0, L4D0, and L5D0), and *HvJHE* (L2D0, L3D0, L4D0, and L5D0) decreased after larval molting and increased during larval feeding. The expression time frames of *HvJHDK*, *HvJHEH*, and *HvJHE* are analogous. The negative correlation between the expression of JH degradation enzymes and the JH titers suggests that these three genes play roles in insect molting by regulating the JH titers. In addition, all three JH degradation genes were highly expressed during the L5 instar stage. Based on these findings, we expect that maintaining high expression levels of these three genes is vital for successful larval–pupal metamorphosis. In holometabolous insects, a pulse of 20 E and high JH titer elicited larval–larval molting, whereas a pulse of 20 E and low JH titer during the final larval instar stage triggered larval–pupal metamorphosis [32]. Furthermore, in the present study, the expression of *HvJHDK*, *HvJHE*, and *HvJHEH* and the titers of JH were higher in adults than in pupae. During larval molting, JH prevents metamorphosis and plays roles in the formation and the development of yolk in adult females. It also plays roles in the development of accessory glands and mating ability in adult males [34].

Suppression of *HvJHDK* increased the JH titers, maintained the increased expression of two JH degradation enzymes (Figure 5), and increased the expression of the two JH response genes *HvMET* and *Hvkr-h1*. The expression of *HvJHAMT* (the last key enzyme in JH synthesis) did not show any significant change (Figure 8). In *Bombyx mori* JHE knockouts, the expression of *JHEH* and *JHDK* increased by 1.8 and 4.1 fold, respectively [19]; in *Nilaparvata lugens*, *Nljhdk* knockdown upregulated *Nljhe* and *Nljheh* expression [35], whereas *Nljheh* knockdown upregulated *Nljhe* expression [21]. Taken together, these findings indicate that there exists a complementary approach in the JH metabolic pathway such that inactivation of one enzyme leads to activation of another. Furthermore, suppression of *HvJHDK* extends the larval duration, and this result is similar to those of *LdJHDK*, *LdJHEH1*, *LdJHEH2* [8,16], and *JHE* knockdown in *Leptinotarsa decemlineata* and *B. mori* [19], which also mediated an abnormal rise in the JH titer in vivo. However, suppression of *HvJHDK* induced an increase in the pupal weights, similar to the effect of *JHE* knockdown but contrary to that of *LdJHDK* knockdown [8]. Abnormal rise in the JH titers may lead to different results between Lepidoptera and Coleoptera. JH may regulate the body size of *H. vitessoides* by mediating the growth duration, as observed in *M. sexta* [36]. 

During the final larval instar stage, the commitment to undergo larval–pupal molting requires the action of ecdysteroids in the absence of JH [37]. After suppression of *HvJHDK*, which induced an abnormal increase of the JH titer, we observed some lethal phenotypes and reduced survival rate in *H. vitessoides*. Previous studies revealed that JH treatment alone suppresses the expression of an epidermal protein associated with cuticle formation [33]. JH also regulates polyphenisms, such as color morphs and caste differentiation [38]. In organisms, enzymes integrate complex metabolic reactions and form an orderly network; changes in the key enzymes directly affect the metabolism of individuals and may even lead to death. Of course, physiological defects in affected insects warrant further research.

Because suppression of *HvJHDK* leads to delayed growth and development, we speculate that *HvJHDK* mediates nutrition and energy metabolism by regulating the JH titers. In this study, TG content decreased in the larval and the pupal stages after 108 and 204 h of injection, respectively (Figure 9). Relative expressions of the lipid accumulation-related gene *HvADH* were decreased, whereas those of the lipid metabolism pathway genes *HvATGL* and *HvTGL* and the lipid degradation-related gene *HvLIP1* were increased. Lipids provide energy for growth during long non-feeding periods and are extremely important for the growth and the reproduction of insects [39]. Our findings suggest that the JH degradation pathway mediated by *HvJHDK* promotes lipid accumulation in both the larval and the pupal stages of *H.* *vitessoides*, and suppression of *HvJHDK* restrains accumulation of TG content. A decrease in TG content resulting in insufficient energy supply during the pupal period could be one of the reasons underlying the low adult emergence rate (53.6% and 38.1% of L4-injected and L5-injected insects, respectively). Furthermore, in *Cnaphalocrocis medinalis*, the TG content was lower in treated adult females than in control adult females 2–4 days after the treatment with JH analog [40]. However, the other JHDK-mediated pathways regulating insect growth need to be further studied.

## 5. Conclusions

In summary, we obtained and identified the *HvJHDK* sequence from the *H. vitessoides* larval transcriptional library. JH titers at a spectrum of developmental stages, expression patterns of *HvJHDK*, *HvJHEH*, and *HvJHE*, and expression of *HvJHDK*, *HvJHEH*, and *HvJHE* after suppression of *HvJHDK* were assessed. Feedback regulation may exist in the JH metabolic pathway. Moreover, 3 and 4 μg/μL were identified as the optimal concentrations for inducing the maximum RNAi efficiency in L4-and L5-injected insects, respectively. Suppression of *HvJHDK* reduced the HvJHDK protein concentration, increasing the JH titer and decreasing the TG content; this may explain the development of extended larval growth, lethal phenotype, and reduced pupation and adult emergence rates. These findings provide a basis for further studying the functions of HvJHDK in the growth and the metabolism of *H. vitessoides*.

## Figures and Tables

**Table 1:** Effect of ds*JHDK* injection on the growth status of *Heortia vitessoides*. CK, controls; L4-injected, ds*JHDK* injected to 12-h-old fourth instar larvae; L5-injected, ds*JHDK* injected to 12-h-old fifth instar larvae. The pupal weight indicated was measured at 1 day of age. Data are presented as mean ± SD (n = 3). The different letters indicate significant differences among the treatments and the control measured at the same time (*p* < 0.05).

	CK	dsGFP	L4-Injected	L5-Injected
Pupal weight (mg)	124.5 ± 2.9ab	119.0 ± 1.9b	130.6 ± 1.9a	128.6 ± 2.6a
L4–L5 survival rate (%)	100 ± 0.00a	98.3 ± 2.89a	83.3 ± 5.77b	
Pupation rate (%)	96.7 ± 2.89a	93.2 ± 3.20ab	82.2 ± 16.08ab	75.0 ± 13.29b
Adult emergence rate (%)	95.0 ± 5.00a	92.8 ± 2.88a	53.6 ± 3.23b	38.1 ± 3.24c

## Supplementary Materials

The following are available online at https://www.mdpi.com/2075-4450/10/9/278/s1, Table S1: PCR primers used in this study; Table S2: GenBank accession number in this study.
