# Supplementary material for: Suppression of Gene Juvenile Hormone Diol Kinase Delays Pupation in Heortia vitessoides Moore
Source: Insects. 2019 Sep 2;10(9):278. doi: 10.3390/insects10090278 (PMC6780227; doi:10.3390/insects10090278)
Supplement: Supplementary file 1 [file insects-10-00278-s001.pdf]

insects

MDPI

Article

Suppression of Gene Juvenile Hormone Diol Kinase Delays Pupation in *Heortia vitessoides* Moore

Zihao Lyu, Zhixing Li, Jie Cheng, Chunyan Wang, Jingxiang Chen and Tong Lin *

College of Forestry and Landscape Architecture, South China Agricultural University, Guangzhou 510642, China

* Correspondence: lintong@scau.edu.cn; Tel.: +86-020-8528-2217

Received: 5 July 2019; Accepted: 25 August 2019; Published: date

Supplementary Materials

Table S1. PCR primers used in this study.

Primers	Primer sequence (5′–3′)
**For real-time PCR**	
HvJHDK-F	AGACACCCTCAACGCAC
HvJHDK-R	ATCTTCTGGAAAGCCGC
HvJHEH-F	GAGAGACAAAGTTGACGAG
HvJHEH-R	CCGAGGAGTGAGAAGAAG
HvJHE-F	CCGGTTTCTCCGACTTCTTCA
HvJHE-R	AGCTTAGCGTTCGCCTCCAT
HvJHAMT-F	AACAAGAACCACAGGGAC
HvJHAMT-R	CGTAGAAGGAGAAAGCGT
HvKr-h1-F	GGTAAACTCCATCGCCA
HvKr-h1-R	AGCACCGCTCTCCGTAA
HvMET-F	ACTTCCTGTTCCACTTGG
HvMET-R	TATTGACTCCTCCGCTGC
HvADH-F	CCACATCCAGTATCCCTTG
HvADH-R	TTTTGGTGCTCCTTCTTTC
HvATGL-F	AAGGGCAGGATCATTGGGT
HvATGL-R	CAGCATTTCAGGCTTGGGA
HvTGL-F	ACCGTTTGTTCGTGTCG
HvTGL-R	CGGAGGTAATAAGTGGCTC
HvLIP1-F	CCTCGTTGACCTGGAAGAA
HvLIP1-R	CCATTGACGGCAGTGTTG
**For ORF**	
JHDK-F	ACGTCTTTACGGTGTTC
JHDK-R	CATTAGCCATCTTCTGG
JHEH-F	GACAGACTACGCACTCG
JHEH-R	CATTCTTCCTACGCCAC
JHE-F	TACTGGACGCCAGCAAAG
JHE-R	CAAACGAGATCCCAAATGC
**For dsRNA**	
dsJHDK-F	TAATACGACTCACTATAGGACACCCTCAACGCACTCTG
dsJHDK-R	TAATACGACTCACTATAGGCCATCTTCTGGAAAGCCGC
dsGFP-F	TAATACGACTCACTATAGGGCAGTTCTTGTTGAATTAGATG
dsGFP-R	TAATACGACTCACTATAGGGTTTGGTTTGTCTCCCATGATG

F: forward primer; R: reverse primer.

insects

MDPI

Table S2. GenBank accession number in this study.

Gene	Genbank number
HvJHDK	MK561746
HvJHEH	MK598043
HvJHE	MK598044
HvJHAMT	MK598045
Hvkr-h1	MK598046
HvMET	MK598047
HvADH	MK598048
HvATGL	MK598049
HvTGL	MK598050
HvLIP1	MH246982
α-tubulin	MG132200
